# The Impact of Bhramari Pranayama and Om Chanting on Post-operative Wound Healing: A Focus on the Nitric Oxide Pathway

**DOI:** 10.7759/cureus.73511

**Published:** 2024-11-12

**Authors:** Saket P Shetty, Aravind v Patil, Jyoti P Khodnapur, Manjunath Kotennavar, Pradeep Jaju, Manjunath Savanth, Veena Ghanteppagol, Narendra Ballal, Divyang GB, Shreeya Doddannavar

**Affiliations:** 1 General Surgery, Shri B. M. Patil Medical College, Hospital and Research Centre, BLDE (Deemed to be University), Vijaypura, IND; 2 General Surgery, Shri B. M. Patil Medical College, Hospital and Research Centre, BLDE (Deemed to be University), Vijayapura, IND; 3 Physiology, Shri B. M. Patil Medical College, Hospital and Research Centre, BLDE (Deemed to be University), Vijayapura, IND; 4 General Surgery, Shri B. M. Patil Medical College, Hospital and Research Centre, BLDE (Deemed to be University), vijaypura, IND

**Keywords:** bhramari pranayama, complementary and alternative medical (cam), heart rate variability (hrv), nitric oxide synthase (enos), om chanting, perceived stress scale (pss), southampton surgical wound assessment scale

## Abstract

Introduction

*Wound healing* is a complex process influenced by various physiological and psychological factors. Stress, in particular, has been shown to impair wound healing by affecting the immune response and slowing the repair process. Yoga-based relaxation techniques, such as bhramari pranayama and Om chanting, have demonstrated the potential to reduce stress and improve overall well-being.

Material and methods

This comprehensive study is a prospective comparative interventional study with a sample size of 268 elective surgery patients. In addition to standard treatment, patients were taught yoga, and various parameters were measured to ensure a thorough evaluation of the impact of these techniques on stress levels and wound healing.

Result

The intervention groups demonstrated significant improvements in wound healing and stress reduction compared to the control group, with the combination of bhramari pranayama and Om chanting showing the most substantial effects. Post-intervention heart rate variability (HRV) low-frequency/high-frequency (LF/HF) ratios decreased to a median of 3.25 (SD 0.14) in the combination group, compared to 4.39 (SD 0.13) in the control group (p<0.001). Endothelial nitric oxide synthase (eNOS) levels increased to 3.49 ng/mL (SD 0.12) from 1.02 ng/mL (SD 0.24) in the control group (p < 0.001). Post-intervention, HRV LF/HF ratios decreased to a median of 3.25 (SD 0.14) in the combination group compared to 4.39 (SD 0.13) in the control group (p<0.001). eNOS levels increased to 3.49 ng/mL (SD 0.12) from 1.02 ng/mL (SD 0.24) in the control group (p < 0.001). Additionally, 36% of participants in the combination group achieved Grade 0 wound healing, compared to 16% in the control group (p<0.001). These findings indicate that yoga interventions effectively enhance post-operative recovery.

Conclusion

This study is pertinent due to the growing interest in integrating complementary and alternative medicine (CAM) with conventional medical practices to enhance patient outcomes. The intersection of yoga-based practices and wound healing presents a novel approach to post-operative care.

## Introduction

A wound is characterized as a disturbance of the integrity of body tissue and its function. Maintenance of the skin's essential protective and regulatory functions depends on its healing ability. It is now well-recognized that yoga-based relaxation and meditation can aid in the healing of wounds by lowering blood sugar, tension, anxiety, and obesity. A handful of studies describe the specific impact of yoga and meditation on healing surgical wounds, indicating that psychological stress can directly or indirectly affect the process of wound healing [1-3].

Routine wound healing occurs in five steps [4]. Hemostasis is initiated by vasoconstriction and platelet plug formation. The inflammatory phase, lasting 2-3 days, involves the release of alpha-granular cytokines, attracting inflammatory mediators like TGF-beta and Platelet-derived growth factor(PDGF), as well as neutrophils and macrophages, to the injury site. After clearing cellular debris and pathogens, the proliferative phase begins in the third week, characterized by fibroblast-mediated production of type III collagen and ground substance, along with angiogenesis and re-epithelialization, leading to granulation tissue formation. This is followed by collagen fiber reorganization, reduced vascular supply, and increased wound contractility.

Various factors determine wound healing. Among them, stress is one factor that profoundly influences the healing process. Stress causes the body's cortisol levels to rise by simultaneously activating the sympathetic-adrenal-medullary (SAM) and hypothalamic-pituitary-adrenal (HPA) axis [5].

Cortisol significantly decreases the circulating titer of white blood cells, their transmigration across the capillary wall, and their chemotaxis. Cortisol also decreases circulating interleukins (IL-1) and other chemokines by inhibiting their production. Growing evidence suggests that yoga-based relaxation can reduce stress, anxiety and depression [2,6].

Yoga may improve wound healing by reducing stress in patients undergoing surgery. According to one study, yoga can help early-stage breast cancer patients heal their wounds and achieve better post-operative results [7].

NO is an important molecule regulating the internal vascular environment, including vascular permeability, vascular compliance, and antithrombotic properties (Palmer RM et al., 1987) [8]. Hence, we aim to study the impact of Om chanting and bhramari pranayama on the healing of wounds and well-being in patients undergoing elective surgery with particular reference to the NO pathway, which plays a crucial role in wound healing by regulating blood flow and promoting tissue repair.

This study holds particular significance as there is a notable gap in the literature concerning the effects of nitric oxide synthase (NOS) on post-operative wound healing, with most existing research limited to animal models, making this investigation crucial for understanding NOS's potential therapeutic implications in enhancing wound healing outcomes in post-operative patients.

## Materials and methods

Source of data

Patients admitted to BLDE (deemed to be university) Shri B.M. Patil Medical College Hospital and Research Centre, Vijayapura, Karnataka, India, a tertiary care center, undergo elective surgeries from the Department of General Surgery, Orthopedic Surgery, and Obstetric and Gynecology. 

Type of study

Prospective comparative interventional study

Study period

The study period extends from July 2022 to June 2024

Ethical consideration

The Institutional Ethics Committee of BLDE (deemed to be a university) issued approval BLDE (DU)/IEC/667/2022-23. The Ethical Committee of this University met on Friday, 26th August 2022, at 3.30 pm in the Department of Pharmacology to scrutinize the synopsis of a Postgraduate Student of BLDE (DU) 's Shri B.M. Patil Medical College Hospital & Research Centre from an Ethical clearance point of view. After scrutiny, the project was accorded ethical clearance.

Sampling

We calculated the sample size using G*Power version 3.1.9.4 software [7]. For the LF/HF (ms²) variable, the baseline study group had a mean of 3.05 (SD = 3.07), and the post-intervention mean was 5.18 (SD = 4.83). This study requires a total sample size of 268 to achieve a power of 99% for detecting a difference in means between two independent groups (t-tests) with a 5% significance level.

Sample size division

A total of 268 patients undergoing elective surgeries were screened based on inclusion and exclusion criteria. Eligible patients were randomly allocated into either the control group or one of three intervention groups. The intervention groups included (1) a Bhramari Pranayama group, (2) a combined Bhramari Pranayama and Om Chanting group, and (3) an Om Chanting group.

Statistical analysis 

The data was entered into a Microsoft Excel spreadsheet, and statistical analysis was performed using Version 20 of the Statistical Package for the Social Sciences (SPSS). The findings are graphs, counts, percentages, mean, and standard deviation. An independent sample t-test will be employed to compare normally distributed continuous variables between the two groups. The Mann-Whitney U test is used for variables that are not regularly distributed. Fisher's exact test, or the Chi-square test, is used to compare categorical variables between the two groups. We have used ANOVA for the Kruskal-Walli H Test for more than two groups, which is not normally distributed. P-values less than 0.05 are deemed statistically significant. Every statistic is run using two tails.

Inclusion and exclusion criteria

Patients with Class I and II wounds [9] who were between the ages of 18 and 59 and were having surgery under general or regional anesthesia were included. Immunocompromised patients or patients on chemotherapy and radiotherapy were excluded.

Methods

Yoga-based meditation: Intervention was given to study group patients for 20 minutes(each yoga exercise) twice daily (morning and evening). Meditation involved bhramari pranayama [10] and Om chanting [11] with closed eyes and focusing on the center of the forehead. To maintain the uniformity of intervention, a certified yoga instructor was appointed. The intervention was started from the first post-operative day onwards till post-operative day 28. Pulse rate and blood pressure (mmHg) were recorded at room temperature between 8 am and 11 am, followed by supine rest for 10 minutes.

Method of collection of data

Assessment Of Stress (Objective Analysis)

Heart rate variability (HRV) was measured to assess stress on post-operative days 1 and 28. In the conventional limb lead II configuration, a five-minute short-term ECG was recorded using a four-channel digital polygraph (Medicaid Systems Pvt. Ltd, Chandigarh, India). The total power was split into three categories: very low frequency (VLF: 0-0.04), low frequency (LF: 0.04-0.15Hz), and high frequency (HF: 0.15-0.40Hz) in the frequency range of 0-0.40Hz. While HF (nu) measures parasympathetic activity, LF (nu) measures mostly reflect sympathetic activity. The LF/HF ratio indicates the balance between the sympathetic and parasympathetic nervous systems.

Assessment Of NO Pathway (Objective Analysis)

Evaluating eNOS levels using the ELISA method, standardized per human endothelial nitric oxide synthase, eNOS GENLISA ELISA (kit REF: KBH0908), on post-operative days 1 and 28.

Assessment Of Wound Healing (Subjective Analysis)

On post-operative days 1 and 28, a wound assessment was done using the Southampton Wound Grading System (Table 1) [12].

**Table 1:** Southampton wound grading system

Grade	Appearance
0	Normal healing
I	Normal healing with mild bruising and/or erythema
Ia	Some bruising
Ib	Considerable bruising
Ic	Mild erythema
II	Erythema and other signs of inflammation
IIa	At only one point
IIb	Around sutures
IIc	Along Wound
IId	Around wound
III	Clear/serous/bloody discharge
IIIa	At only one point (= 2cm)
IIIb	Along the wound (> 2cm)
IIIc	Large volume
IIId	Prolonged (> 3 days)
Major complication	
IV	Pus
Iva	At one point only (= 2cm)
IVb	Along wound (>2cm)
V	Deep/Severe wound infection with or without tissue

 *Assessment Of Stress ( Subjective Analysis)*

Stress assessment was done using perceived stress scale questionnaire assessment done on day one and day twenty-eight of the postoperative day [13].

## Results

Table 2 demonstrates a well-balanced age distribution among patients across four groups. Most participants were in the 18-29 age group, accounting for 39-43% of each group. The 30-39 age group comprised 13-24% of patients, while 18-22% were aged 40-49. The 50-59 age group comprised 18-25% of the patients. This distribution reflects a broad representation of adult age ranges in the study population.

**Table 2:** Age distribution under each group n(%)- number of patients (percentage of patients)

Age (Years)	Bhramari pranayama, n (%)	Bhramari pranayama and Om chanting, n (%)	Om chanting, n (%)	Control, n (%)
18-29	26 (39%)	26 (39%)	29 (43%)	28 (42%)
30-39	15 (22%)	16 (24%)	10 (15%)	9 (13%)
40-49	9 (13%)	13 (19%)	12 (18%)	15 (22%)
51-59	17 (25%)	12 (18%)	16 (24%)	15 (22%)

Table 3 shows how patients were divided into four groups according to the kind of operation. Across all categories, general surgery were the most common, with 22% to 27% of patients having these procedures. Of the patients, 22% to 31% underwent orthopedic procedures, and 24% to 25% underwent obstetrics and gynecology (OB/GYN) surgeries. This distribution shows that the study groups’ surgical procedures varied consistently.

**Table 3:** Distribution of the patients based on the type of intervention they underwent n(%)- number of patients(percentage of patients)

Type of procedure	Bhramari pranayama, n(%)	Bhramari pranayama and Om chanting, n(%)	Om chanting, n(%)	Control, n(%)
OB/GYN	25 (24%)	26 (25%)	26 (25%)	26 (25%)
ORTHOPAEDIC	11 (22%)	16 (31%)	13 (25%)	11 (22%)
GENERAL SURGERY	31 (27%)	25 (22%)	28 (25%)	30 (26%)

The demographic analysis of the study population showed that most female patients were either homemakers or students. On the other hand, many male patients were daily wage workers or farmers. This distribution corresponds to the socio-economic background of the region, emphasizing the main occupational roles and responsibilities of each gender in this study. The different work demands between these groups may also have implications for stress levels and postoperative recovery.

Table 4 shows the Heart rate variability (LF/HF ratio). Post-intervention, the HRV values show distinct changes: the Control group’s HRV values are more or less the same, with increased enhanced sympathetic activity. Conversely, the intervention groups exhibit decreased HRV values, suggesting a reduction in the LF/HF ratio and increased parasympathetic activity. The significant P values (<0.001) from the Kruskal-Wallis tests for HRV pre- and post-confirm that these differences are statistically significant and not due to random variation.

**Table 4:** Pre-intervention & post-intervention heart rate variability (Low Frequency/High Frequency) levels IQR: Inter quartile range; HRV: Heart rate variability; LF: Low-frequency; HF: High-frequency *P value <0.05 is considered statistically significant

Heart rate variability	Brahmari pranayama , Median (IQR)	Brahmari pranayama and Om chanting, Median (IQR)	Om chanting, Median (IQR)	Control, Median (IQR)	P-Value
HRV pre(LF/HF ratio)	4.28 (4.17, 4.51)	4.26 (4.16, 4.40)	4.14 (4.04, 4.23)	4.24 (4.13, 4.38	<0.001*
HRV post(LF/HF ratio)	3.14 (3.04, 3.23)	3.25 (3.16, 3.43)	3.15 (3.08, 3.40)	4.39 (4.21, 4.49)	

Table 5 illustrates the impact of yoga on stress levels. Post-intervention analysis reveals significant shifts. While the control and intervention groups initially exhibited high-stress levels, the control group saw a marked increase in moderate stress. In contrast, the intervention group experienced a significant increase in the percentage of participants reporting low stress. There is a statistically significant difference between the control and intervention groups (P-value < 0.001).

**Table 5:** Pre-intervention & post-intervention stress level n(%)- number of patients(percentage of patients) *P value <0.05 is considered statistically significant

Stress levels	Bhramari pranayama, n(%)	Bhramari pranayama and Om chanting, n(%)	Om chanting, n(%)	Control, n(%)	p-value
Pre-intervention Stress Scale					<0.001*
High Stress	15 (16%)	21 (23%)	10 (11%)	46 (50%)	
Moderate stress	52 (30%)	46 (26%)	57 (32%)	21 (12%)	
Post-intervention Stress Scale					
Low Stress	57 (32%)	55 (31%	54 (31%)	10 (5.7%)	
Moderate Stress	10 (11%)	12 (13%)	13 (14%)	57 (62%)	

Table 6 shows the distribution of wound categories before and after the intervention. The analysis indicates significant differences in wound severity between the control and intervention groups. The intervention groups showed notable improvement, as reflected by higher percentages in the less severe wound categories (1A, 1B, 1C) and the appearance of participants with no wounds (Category 0). In contrast, the Control group had consistently higher percentages of severe wounds (Categories 3A, 3B, 3C, 4A, 4B, 4D, and 5) before and after the intervention with statistical significance of these findings, with p-values <0.001.

**Table 6:** Pre-intervention and Post-intervention wound scoring n(%)- number of patients(percentage of patients) *P value <0.05 is considered statistically significant

Wound Assessment	Brahmari pranayama, n(%)	Brahmari pranayama and Omkar chanting, n(%)	Om chanting, n(%)	Control, n(%)	p-value
Pre-intervention wound assessment					<0.001*
0	0 (0%)	1 (100%)	0 (0%)	0 (0%)	
1A	12 (33%)	8 (22%)	13 (36%)	3 (8.3%)	
1B	9 (31%)	6 (21%)	9 (31%)	5 (17%)	
1C	19 (24%)	20 (26%)	20 (26%)	19 (24%)	
2A	8 (33%)	4 (17%)	6 (25%)	6 (25%)	
2B	8 (27%)	7 (23%)	7 (23%)	8 (27%)	
2C	4 (22%)	4 (22%)	4 (22%)	6 (33%)	
2D	2 (5.0%)	14 (35%)	7 (18%)	17 (43%)	
3A	4 (40%)	3 (30%)	1 (10%)	2 (20%)	
3B	0 (0%)	0 (0%)	0 (0%)	1 (100%)	
3C	1 (100%)	0 (0%)	0 (0%)	0 (0%)	
Post-intervention wound assessment					
0	17 (28%)	22 (36%)	12 (20%)	10 (16%)	
1A	19 (25%)	26 (35%)	20 (27%)	10 (13%)	
1B	3 (12%)	7 (28%)	10 (40%)	5 (20%)	
1C	11 (37%)	6 (20%)	9 (30%)	4 (13%)	
2A	10 (42%)	1 (4.2%)	8 (33%)	5 (21%))	
2B	2 (18%)	1 (9.1%)	2 (18%)	6 (55%)	
2C	0 (0%)	0 (0%)	0 (0%))	1 (100%)	
2D	1 (33%)	0 (0%)	0 (0%)	2 (67%)	
3A	1 (8.3%)	0 (0%)	0 (0%)	11 (92%)	
3B	0 (0%)	1 (13%)	4 (50%)	3 (38%)	
3C	0 (0%)	0 (0%)	0 (0%)	1 (100%)	
3D	0 (0%)	2 (40%)	0 (0%)	3 (60%)	
4A	1 (20%)	1 (20%)	1 (20%)	2 (40%)	
4B	1 (33%)	0 (0%)	0 (0%)	2 (67%)	
4D	0 (0%)	0 (0%)	0 (0%)	1 (100%)	
5	1 (33%)	0 (0%)	1 (33%)	1 (33%)	

Table 7 shows the effect of intervention on endothelial nitric oxide synthase levels (eNOS). The analysis reveals significant post-intervention changes in eNOS values, with the intervention group demonstrating marked increases. These increases are statistically significant, as indicated by a P value <0.001. In contrast, the Control group showed no substantial change in eNOS values. These findings suggest that the interventions are effective in enhancing eNOS production.

**Table 7:** Pre-intervention and post-intervention endothelial nitric oxide synthase IQR: Inter Quartile Range; eNOS: Endothelial nitric oxide synthase *P value <0.05 is considered statistically significant

eNOS	Brahmari pranayama, Median(IQR)	Brahmari pranayama and Om chanting, Median(IQR)	Om chanting, Median(IQR)	Control, Median(IQR)	p-value
Pre-intervention	1.11 (0.63, 1.45)	0.98 (0.60, 1.56)	0.98 (0.60, 1.39)	1.27 (0.75, 1.51)	<0.001*
Post-intervention	3.41 (3.16, 3.61)	3.49 (3.12, 3.61)	3.34 (3.19, 3.50)	1.02 (0.72, 1.20)	

Of the 268 patients initially enrolled, 87 were excluded from the study, with the majority of exclusions resulting from loss to follow-up. To preserve the intended sample size, additional patients were recruited to replace those excluded. For each patient lost to follow-up, a new participant was enrolled, ensuring that the total number of cases remained stable throughout the duration of the study.

## Discussion

In the present study, we conducted comprehensive assessments of patients wound healing and stress levels during the post-operative period. This was done through both objective measures, such as the perceived stress scale and the Southampton wound grading system, and subjective measures, such as heart rate variability and endothelial nitric oxide synthase (eNOS).

Stress was evaluated subjectively through the perceived stress scale and objectively by HRV levels following the yoga intervention. Balvinder Kaur et al. demonstrated that HRV features effectively distinguished between normal and stressed conditions, with both logistic regression and linear discriminant analysis classifiers performing well. The area under the curve ranged from 0.63 to 0.76, with optimal performance during the most stressful condition (delivering a speech), achieving sensitivity and accuracy above 60%. These findings support using HRV-driven classifiers for stress detection in various applications [14]. The results of this study showed significant improvements in both HRV and stress levels with P value <0.05. The strong correlation between these outcomes further reinforces the effectiveness of the intervention.

Erica Sharpe et al. (2021) conducted A study that found that both externally paced and self-paced pranayama practices significantly increased heart rate variability (HRV), as evaluated by root mean square of successive differences (RMSSD). The results indicate pranayama's favorable effect on cardiac parasympathetic tone, showing that it can reduce stress and regulate autonomic function [15]. Heart rate variability (HRV) was investigated by Claudia Arab et al. (2016) in relation to various stages of breast cancer [16]. The results showed that patients with recently discovered breast cancer had abnormal heart rate variability (HRV), which was indicative of disturbed autonomic function. Similarly, patients experiencing adverse effects from breast cancer treatment showed comparable autonomic disturbances. Conversely, those in the recovery phase demonstrated notable improvements in HRV, suggesting a restoration of autonomic balance. These findings are consistent with our results and establish a connection between stress and wound healing.

Wound healing was evaluated subjectively by the Southampton wound grading system and objectively by endothelial nitric oxide levels. Nitric oxide (NO) is critical in normal wound healing, as evidenced by various studies involving genetically modified mice and pharmacological interventions. A study conducted by Yamasak K et al. demonstrated that inducible nitric oxide synthase (iNOS) plays a critical role in wound healing [17]. The study found that mice deficient in iNOS showed significantly delayed wound closure compared to wild-type mice. This delay was also observed when wild-type mice were treated with an iNOS inhibitor. However, when an adenoviral vector carrying the human iNOS gene was applied, the impaired healing in iNOS-deficient mice was reversed, promoting normal wound closure. These findings suggest that iNOS is essential for effective wound healing and may have therapeutic potential for treating impaired wound-healing conditions. Paul C. Lee et al. found that endothelial nitric oxide synthase (eNOS) is critical in wound healing and angiogenesis [18]. eNOS knockout (KO) mice exhibited significantly delayed wound closure and reduced tensile strength in healing incisions compared to wild-type mice. In-vitro assays revealed impaired endothelial cell migration, proliferation, and differentiation in eNOS KO mice, while in-vivo angiogenesis assays showed a marked reduction in capillary formation. These results suggest that eNOS is essential for effective wound healing and growth factor-stimulated angiogenesis. These animal models underscore the critical role of nitric oxide synthase (NOS) in wound healing. Both studies highlight the importance of iNOS and eNOS in facilitating proper wound closure and angiogenesis. The findings from Yamasaki K et al. and Lee PC et al. demonstrate that deficiencies in iNOS or eNOS significantly impair wound healing, supporting the essential function of NOS in tissue repair. Our findings of elevated eNOS levels in the post-interventional period further correlate with enhanced wound healing, with a significant p-value of <0.05, reinforcing the pivotal role of eNOS in promoting effective wound healing and angiogenesis.

Taneja MK et al. stated that Humming (bhramari pranayama) significantly boosts nitric oxide production in the sinuses and nasal mucosa. This activity is estimated to increase the endogenous generation of nitric oxide levels by up to 15 times compared to quiet exhalation [19]. Rao Raghavendra M. et al. noted yoga's impact on people with breast cancer that is operable but in its early stages [7]. The results show that the yoga intervention lowered the time between drain and suture removals and hospital admissions. Patients initially under stress throughout the recovery phase had higher cortisol levels and slower wound healing. Malone-Povolny MJ et al. review the role of nitric oxide (NO) in diabetic wound healing and its potential as a therapeutic agent [20]. The findings suggest that NO-based treatments can enhance wound healing, particularly in diabetic patients, by improving collagen deposition, re-epithelization, and angiogenesis while also controlling infection. This progress highlights the therapeutic potential of NO for chronic wound management. Alexandra Igrunkova et al. demonstrated that the application of a nitric oxide (NO)-donor spray, specifically a dinitrosyl iron complex (DNIC), significantly accelerated wound healing [21]. The spray with a 50 µg dosage of DNIC showed the most beneficial effects, including a 140% increase in granulation tissue thickness, reduced inflammation, and enhanced collagen maturation compared to the control group. Victoria AZ et al. demonstrated that nitric oxide (NO) therapy, delivered through the plasma-chemical device "Plason," significantly accelerated the healing of both skin and muscle injuries in a rat model and professional soccer players with bruises [22]. In rats, NO therapy reduced inflammation and promoted the maturation of granulation tissue. In soccer players, the treatment led to faster pain reduction, resorption of hematomas, and reduced edema compared to the control group. Athletes treated with NO therapy returned to full training sooner, highlighting the potential of NO therapy in accelerating recovery from sports injuries. The findings of these studies support ours, showing that pranayama-induced elevation of eNOS and reduction of stress are associated with improved wound healing, with a p-value of less than 0.05.

This study provides valuable insights into the positive effects of bhramari pranayama and om chanting on post-operative wound healing and stress reduction; however, several limitations should be considered. The study's sample size of 268 patients, while statistically significant, was derived from a single institution, limiting the generalizability of the findings. Future multicenter studies with more diverse populations are needed to validate these results. Additionally, the follow-up period of 28 days may not fully capture the long-term effects of these interventions on wound healing and stress recurrence. The reliance on self-reported stress measures, such as the Perceived Stress Scale, introduces subjectivity, although objective measures like heart rate variability (HRV) were used. Incorporating more objective biomarkers in future studies could enhance the accuracy of stress evaluation.

Furthermore, excluding immunocompromised patients or those undergoing chemotherapy and radiotherapy limits the findings' applicability to patients with more complex medical conditions. Lastly, variations in patient adherence to the interventions, despite instructor guidance, may have influenced the outcomes, as the fidelity to the techniques was not closely monitored. Addressing these limitations in future research could provide a more comprehensive understanding of the interventions' full potential across various clinical settings.

## Conclusions

This study highlights the significant impact of yoga-based interventions, particularly the combination of bhramari pranayama and om chanting, on enhancing post-operative wound healing and reducing stress. The interventions yielded improvements across subjective and objective measures: wound healing assessed through the Southampton wound grading system and stress through the perceived stress scale, complemented by objective measures, including eNOS levels and heart rate variability (HRV). The alignment between subjective assessments and objective data strengthens the validity of our findings. Through correlating these insights, we conclude that incorporating such complementary practices into standard post-operative care could significantly support patient recovery and optimize outcomes. Adopting these holistic approaches in clinical settings as part of comprehensive post-operative management could address wound healing and the stress factors that negatively influence recovery.

## References

[1] Lucas VS (2011). ﻿﻿﻿﻿﻿﻿﻿﻿﻿﻿﻿﻿﻿﻿Psychological stress and wound healing in humans: What we know. Wounds.

[2] Smith C, Hancock H, Blake-Mortimer J, Eckert K (2007). A randomised comparative trial of yoga and relaxation to reduce stress and anxiety. Complement Ther Med.

[3] Reza T, Grezenko H, Barker C (2023). Emotional stress and immune response in surgery: A psychoneuroimmunological perspective. Cureus.

[4] Gonzalez AC, Costa TF, Andrade ZA, Medrado AR (2016). Wound healing - A literature review. An Bras Dermatol.

[5] Yang E V, Glaser R (2002). Stress-induced immunomodulation and the implications for health. Int Immunopharmacol.

[6] Patil SG, Mullur LM, Khodnapur JP, Dhanakshirur GB, Aithala MR (2013). Effect of yoga on short-term heart rate variability measure as a stress index in subjunior cyclists: A pilot study. Indian J Physiol Pharmacol.

[7] Rao RM, Nagendra HR, Raghuram N, Vinay C, Chandrashekara S, Gopinath KS, Srinath BS (2008). Influence of yoga on postoperative outcomes and wound healing in early operable breast cancer patients undergoing surgery. Int J Yoga.

[8] Radomski MW, Palmer RM, Moncada S (1987). The anti-aggregating properties of vascular endothelium: Interactions between prostacyclin and nitric oxide. Br J Pharmacol.

[9] Abuihmoud A, Linchangco P, Wise E, Boldyga A, Nachman K, Gomez BT, Parada JP (2018). 2136. Systematic review of surgical wound class reveals marked service-related discrepancies and can improve appropriateness of classification impacting the expected number of Infections and the standardized infection ratio (SIR). Open Forum Infect Dis.

[10] Chetry D, Telles S, Mahadevan J, Prasoon K, Gandharva K, Agrawal M, Balkrishna A (2022). A comparison of practice guidelines for yoga breathing from the traditional texts and pubmed-indexed research. Int J Yoga Therap.

[11] Inbaraj G, Rao RM, Ram A (2022). Immediate effects of om chanting on heart rate variability measures compared between experienced and inexperienced yoga practitioners. Int J Yoga.

[12] Gorad K, Rahate V, Shinde G, Taralekar G, Prabhu V, Singh L (2021). Use of southampton scoring for wound healing in post-surgical patients: Our experience in semi-urban setup. Arch Clin Biomed Res.

[13] Lee EH (2012). Review of the psychometric evidence of the perceived stress scale. Asian Nurs Res (Korean Soc Nurs Sci).

[14] Kaur B, Durek JJ, O'Kane BL, Tran N, Moses S, Luthra M, Ikonomidou VN (2014). Heart rate variability (HRV): An indicator of stress. SPIE Proceedings.

[15] Sharpe E, Lacombe A, Sadowski A (2021). Investigating components of pranayama for effects on heart rate variability. J Psychosom Res.

[16] Arab C, Dias DP, Barbosa RT (2016). Heart rate variability measure in breast cancer patients and survivors: A systematic review. Psychoneuroendocrinology.

[17] Yamasaki K, Edington HD, McClosky C (1998). Reversal of impaired wound repair in iNOS-deficient mice by topical adenoviral-mediated iNOS gene transfer. J Clin Invest.

[18] Lee PC, Salyapongse AN, Bragdon GA, Shears LL 2nd, Watkins SC, Edington HD, Billiar TR (1999). Impaired wound healing and angiogenesis in eNOS-deficient mice. Am J Physiol.

[19] Taneja MK (2016). Nitric oxide bhramari pranayam and deafness. Indian J Otol.

[20] Malone-Povolny MJ, Maloney SE, Schoenfisch MH (2019). Nitric oxide therapy for diabetic wound healing. Adv Healthc Mater.

[21] Igrunkova A, Fayzullin A, Churbanov S (2022). Spray with nitric oxide donor accelerates wound healing: Potential off-the-shelf solution for therapy?. Drug Des Devel Ther.

[22] Zaborova VA, Butenko AV, Shekhter AB (2023). Nitric oxide therapy is beneficial to rehabilitation in professional soccer players: Clinical and experimental studies. Med Gas Res.

